# Improving the use of research evidence in guideline development: 11. Incorporating considerations of cost-effectiveness, affordability and resource implications

**DOI:** 10.1186/1478-4505-4-23

**Published:** 2006-12-05

**Authors:** Tessa Tan-Torres Edejer

**Affiliations:** 1 Health Systems Financing, World Health Organization, CH-1211 Geneva 27, Switzerland

## Abstract

**Background:**

The World Health Organization (WHO), like many other organisations around the world, has recognised the need to use more rigorous processes to ensure that health care recommendations are informed by the best available research evidence. This is the 11^th ^of a series of 16 reviews that have been prepared as background for advice from the WHO Advisory Committee on Health Research to WHO on how to achieve this.

**Objectives:**

We reviewed the literature on incorporating considerations of cost-effectiveness, affordability and resource implications in guidelines and recommendations.

**Methods:**

We searched PubMed and three databases of methodological studies for existing systematic reviews and relevant methodological research. We did not conduct systematic reviews ourselves. Our conclusions are based on the available evidence, consideration of what WHO and other organisations are doing and logical arguments.

**Key questions and answers:**

When is it important to incorporate cost-effectiveness, resource implications and affordability considerations in WHO guidelines (which topics)?

• For cost-effectiveness:

The need for cost/effectiveness information should be dictated by the specific question, of which several may be addressed in a single guideline. It is proposed that the indications for undertaking a cost-effectiveness analysis (CEA) could be a starting point for determining which recommendation(s) in the guideline would benefit from such analysis.

• For resource implications/affordability:

The resource implications of *each *individual recommendation need to be considered when implementation issues are being discussed.

How can cost-effectiveness, resource implications and affordability be explicitly taken into account in WHO guidelines?

• For cost-effectiveness:

∘ If data are available, the ideal time to consider cost-effectiveness is during the evidence gathering and synthesizing stage. However, because of the inconsistent availability of CEAs and the procedural difficulty associated with adjusting results from different CEAs to make them comparable, it is also possible for cost-effectiveness to be considered during the stage of developing recommendations.

∘ Depending on the quantity and quality and relevance of the data available, such data can be considered in a qualitative way or in a quantitative way, ranging from a listing of the costs to a modelling exercise. At the very least, a qualitative approach like a commentary outlining the economic issues that need to be considered is necessary. If a quantitative approach is to be used, the full model should be transparent and comprehensive.

• For resource implications/affordability:

∘ Resource implications, including health system changes, for each recommendation in a WHO guideline should be explored. At the minimum, a qualitative description that can serve as a gross indicator of the amount of resources needed, relative to current practice, should be provided.

How does one provide guidance in contextualizing guideline recommendations at the country level based on considerations of cost-effectiveness, resource implications and affordability?

• All models should be made available and ideally are designed to allow for analysts to make changes in key parameters and reapply results in their own country.

• In the global guidelines, scenarios and extensive sensitivity/uncertainty analysis can be applied.

Resource implications for WHO

• From the above, it is clear that guidelines development groups will need a health economist. There is need to ensure that this is included in the budget for guidelines and that there is in-house support for this as well.

## Background

The World Health Organization (WHO), like many other organisations around the world, has recognised the need to use more rigorous processes to ensure that health care recommendations are informed by the best available research evidence. This is the 11^th ^of a series of 16 reviews that have been prepared as background for advice from the WHO Advisory Committee on Health Research to WHO on how to achieve this. In this paper we address the following questions:

• When is it important to incorporate cost-effectiveness, resource implications and affordability considerations in WHO guidelines (which topics)?

• How can cost-effectiveness, resource implications and affordability be explicitly taken into account in WHO guidelines?

• How does one provide guidance in contextualizing guideline recommendations at the country level based on considerations of cost-effectiveness, resource implications and affordability?

• What are the resource implications of the answers to these questions for WHO?

## What is WHO doing now?

In 2003, the Guidelines for WHO Guidelines [1] recommended that both cost-effectiveness and resource implications of guideline recommendations be considered when developing WHO guidelines. With the primary audience being Ministry of Health officials with a mandate to improve population health rather than the health of individuals, such concerns were considered to be appropriate. The guidelines (see Additional file 1 for relevant portions) states very briefly that cost-effectiveness is to be considered during the stage of formulation of the recommendations and that the recommendations could be expressed in terms of scenarios (for countries with limited versus some resources). It also recommends that 1–2 experts in cost-effectiveness analysis (CEA) be involved in the technical guidelines development group. Finally, in the third stage or the localization phase in-country, it suggests that WHO provide technical assistance for countries to use their own or regional cost-effectiveness information and that WHO also provide information on financial costs of implementing the recommendations.

There has been some mention of cost-effectiveness in a few guidelines that were issued since 2003 and that claim to have used the Guidelines for WHO Guidelines. The 2003 WHO/ISH guidelines [2] had general sections on cost-effectiveness and resource implications of the recommendations. More recently, the WHO "guidelines" on hand hygiene in health care [3] also included information on economic burden of poor hygiene, cost-effectiveness and resource implications of recommendations. Another example, the malaria treatment guidelines [4] specifically state that cost-effectiveness studies were not included for consideration because there were very few completed studies relating to the interventions being considered, and that the prices of the anti-malarial drugs were fluid, "rendering such studies unreliable."

## What other organizations are doing?

It is clear that cost-effectiveness and/or cost implications of recommendations are recognized in guideline development. The US Preventive Services Task Force [5] lists several reasons why CEA is useful in guideline development:

1. Quantifying the differences between two or more effective services for the same condition

2. Illustrating the impact of delivering a given intervention at different intervals, different ages, or to different risk groups

3. Evaluating the potential role of new technologies

4. Identifying key conditions that must be met to achieve the intended benefit of an intervention

5. Incorporating preferences for intervention outcomes

6. Developing a ranking of services in order of their costs and expected benefits

The AGREE guidelines appraisal instrument [6], on which the WHO checklist was loosely based, includes the cost impact of guideline recommendations under their applicability criteria.

To actually gauge what other organizations are doing with respect to cost-effectiveness/costs in guideline development, one can review the guidelines that have been issued by these organizations and/or review their documented methods for guideline development. It is hoped that there is consistency between the two. In 1999, 279 guidelines that were published in peer-reviewed literature were reviewed [7]. Only 41.6% made any mention of projected effects on health care costs, and only 14.3% quantified these estimates in any way. A 2002 study on the incorporation of published cost-effectiveness analysis in published clinical guidelines showed that, using guidelines as the unit of analysis, 9 of 35 (26%) incorporated at least 1 economic analysis of above-average quality in the text and 11 of 35 (31%) incorporated at least 1 in the references [8]. Finally, a search of the database of the National Guideline Clearinghouse [9] showed that of 1616 guidelines published between 2000–2005, only 369 or 23% had a formal cost analysis.

For a review of methods, a 2003 survey [10] of 18 clinical practice guideline development agencies showed that six included costs/cost containment/cost-effectiveness in their objectives but only three routinely included health economists in their guideline development groups (NHRMC in Australia, SBU in Sweden, and North of England). The AGREE prototype electronic library shows some illustrative excerpts specifically mentioning costs from the methodologies of five national agencies. More detailed information on the use of costs/cost-effectiveness information by guidelines agencies is available (see Additional file 2).

To what extent these organizations routinely and explicitly use costs/CEA in their guidelines is not clear. More information is needed on the actual experience of these organizations in incorporating costs/cost-effectiveness information during their guideline development process (e.g. lessons learned) [11] and an assessment of whether economic evaluations have provided added value to their guidelines [12].

## Methods

The methods used to prepare this review are described in the introduction to this series [13]. Briefly, the key questions addressed in this paper were vetted amongst the authors of the series of articles and the ACHR Subcommittee on the Use of Research Evidence (SURE). We did not conduct full systematic reviews. We searched PubMed and three databases of methodological studies (the Cochrane Methodology Register, the US National Guideline Clearinghouse, and the Guidelines International Network for existing systematic reviews and relevant methodological research that address these questions. The answers to the questions are our conclusions based on the available evidence, consideration of what WHO and other organisations are doing, and logical arguments.

For this review PubMed was searched using the following text word searches: costs and generalizability, practice guidelines and cost-effectiveness analysis and combinations thereof. Using the same search words, the Internet search machine, Google, was also used to search for unpublished documents. Websites of known repositories of guidelines, of organizations of guideline agencies, and of pioneer/well-known guideline development agencies were visited. References in key documents that had titles which could be probably relevant were also pursued. A few times, when it was evident that an author had this as his/her special area of interest, a search using his/her name in connection with guidelines was also done. Papers were included if they described guidelines that included cost-effectiveness information, or described methods of guideline agencies to incorporate costs/resource implications/CEA information.

The information collected during the review was then synthesized where relevant for each question and was used as the basis to draw the implications for WHO guidelines.

## Findings

### When is it important to incorporate considerations of cost-effectiveness and resource implications of recommendations in WHO guidelines (which topics)?

The need for cost-effectiveness information should be dictated by the specific question of which several may be addressed in one guideline alone. It is proposed that the indications for undertaking a cost-effectiveness analysis could be a starting point for determining which recommendation(s) in the guideline would benefit from such analysis [14]. From the review, NHRMC states explicitly that "the challenge is to focus on the decision points that are of key importance in an economic sense and pinpoint the nature of economic information needed to address these questions. The key decisions concern health care that contributes significantly to the total cost of an option, options with very different costs or care that contributes significantly to health outcomes. On the other hand, decisions are unimportant if they concern health care that is uncontroversial, options that are not economically viable or options for which there are no large resource implications." [15]

The UK National Institute for Health and Clinical Excellence (NICE) extends this concept by adopting a value of analysis approach where aside from "the overall 'importance' of the recommendation (which is a function of the number of patients affected and the potential impact on costs and health outcomes per patient)", they also suggest evaluating "the current extent of uncertainty over cost-effectiveness and the likelihood that analysis will reduce this uncertainty" [16].

#### Implications for WHO guidelines

At the scoping stage, the assistance of an experienced health economist who is familiar with the area of interest would be needed and a selective identification of the issues needing CEA could be done. If CEA is not initially identified during the scoping stage as a clear need, this issue needs to be revisited again at the evidence, recommendations and peer review stage.

On the other hand, the resource implications of *each *individual recommendation needs to be considered when implementation issues are being discussed. This may be done at the global guideline level, through the use of scenarios, and at the local adaptation or country level.

### How can cost-effectiveness and affordability be explicitly taken into account in WHO guidelines?

There are different points in the guideline development process that cost-effectiveness and resource implications of guidelines recommendations can be considered. The first phase is the evidence phase and to the extent possible, this is where cost-effectiveness information should be considered.

As in questions on effectiveness, the question can be raised as to why conduct a review of CEAs rather than identify a single study that addresses the question? "CEAs vary widely in their methods and assumptions. Because of this variation, systematically reviewing CEAs provides several benefits. First, because CEAs draw on a variety of cost and effectiveness data sources to develop input parameters, a systematic review can identify which analyses use the best available evidence for key inputs and are therefore the most evidence based. Second, because the credibility of CEAs rests on their quality, a critical review of CEAs and a rating of the quality of each allow for identifying the most methodologically rigorous studies. Third, a comprehensive review can identify the studies that best address the question being asked. Fourth, comparatively assessing CEAs can help to identify variables and methods that significantly influence the estimated benefits and cost effectiveness of an intervention. For instance, some CEAs might assume no harms from a given intervention, while others might assume that the intervention has significant harms. Comparing these studies side by side may provide insight into how the assumption or lack of assumption of harm affects the estimated benefit of the intervention. While some assumptions are varied within a single study using sensitivity analysis, most CEAs provide a limited number of sensitivity analyses. Thus, systematically reviewing CEAs may help identify, through a side-by-side comparison that amounts to a "virtual sensitivity analysis," the impact of different assumptions on the benefits of a given intervention. Finally, the more high-quality, independently conducted CEAs there are for a given intervention, the more convincing the evidence." [17]

In doing a systematic review of economic evaluations, the first step is to search for the literature. There are available resources on the internet which list databases and compilations of economic evaluations [18]. In addition, there has been a systematic evaluation in terms of sensitivity and specificity of different search strategies for economic evaluations [19].

In extracting data, considerable progress has been reported with development of a systematic process of adjustments of results from different studies to make them comparable. The Task Force on Community Preventive Services admits that "no process of adjustment or other means of reviewing existing economic evaluations is flawless." But it makes the point that to adjust data to make it comparable is better than to "(1) ignore economic information entirely; (2) attempt to use non-comparable data; or (3) adjust in ways that are not systematic or explicit" [20].

Some work has also been done in terms of very simple visual methods to present summaries of cost-effectiveness analysis [21]. Despite all of these advances methodologically, the current situation shows however, that there is limited availability and variable quality of relevant CEAs [22].

#### Implications for WHO guidelines

If data is available, **t**he ideal time to consider CEA is during the evidence gathering and synthesizing stage. However, because of the inconsistent availability of CEAs and the procedural difficulty associated with adjusting results from different CEAs to make them comparable [20] it is also possible for CEA to be considered during the stage of developing recommendations. This is also consistent with the GRADE approach [23]. At this stage, the information of the resource implications and outcomes of the recommended interventions can be considered simultaneously. Depending on the quantity and quality and relevance of the data available, such data can be considered in a qualitative way or in a quantitative way, ranging from a listing of the costs to a modelling exercise [24,25]. At the very least, a qualitative approach like a commentary outlining the economic issues that need to be considered is necessary [26]. If a quantitative approach is to be used, the full model should be transparent, be made available and extensive uncertainty/sensitivity analysis built-in so as to allow analysts to selectively reapply results in their own country, as in the WHO-CHOICE contextualization tool [27].

### How does one provide guidance in contextualizing guideline recommendations at the country level based on considerations of cost-effectiveness and affordability?

For cost-effectiveness, there are concerns about the generalizability of results from a single CEA or even a systematic review of a CEA. A review of sources of variability frequently mentions volume and costs of resources consumed as a source of variability [28]. Not as much work has been done on variability of outcomes. Very recently, a checklist was developed for assessing variability or generalizability to be able to translate information from one developed country to another [29]. There is a need to pilot test this, a revision or another instrument in developing countries. For costs, more specifically prices, general principles for adaptation are available [30].

Affordability or resource implications can be considered in the global guidelines if it gives guidance by provision of basic information that will allow guideline users to work out the cost implications for their own service [25,15]. A scenario approach can be used. Also, in this context, WHO-CHOICE data and methods are useful for contextualization [31]. Note that this exercise will also need to include the health system implications of the recommendations, from training, changes in supervision, monitoring and evaluation, advocacy, etc. as seen in some recent examples [32,33].

#### Implications for WHO

Resource implications, including health system changes, for each recommendation in WHO guidelines should be explored. At the minimum, a qualitative description that can serve as a gross indicator of the amount of resources needed, relative to current practice, should be provided.

### Overall assessment of need for health economics expertise

In summary, the role of the health economist in a guideline development group is to:

• help to identify the clinical issues or questions for economic analysis

• review economic literature

• carry out or commission cost-effectiveness analyses

• estimate the cost and resource implications of the recommendations.

"The relative weight given to each role will vary from guideline to guideline. There may be large differences between guidelines in respect of the literature available to review: the size of the relevant economics literature, its relevance, its quality, its timeliness, its generalisability. In some areas there may be good-quality data that can be used in economic models, whereas other areas may have a dearth of such data." [16] Additionally, all throughout the process, the health economist can educate the other guideline development group about CEA and vice-versa, through interaction with the guideline development group members, s/he will be better able to work with an improved understanding of the health issues being considered.

#### Implications for WHO

From the above, it is clear that guidelines development groups will need access to a health economist. There is need to ensure that this is budgeted for in the resources and that there is in-house support for this as well.

## Further work

There is a need to: 1) get more information on the actual experience of guideline agencies in incorporating CEA in guidelines; 2) assess the added value of economic evaluations in guidelines by comparing recommendations with and without CEA; 3) further expand the section on CEA and resource implications in guidelines, including specification of the minimum information that should be provided, in collaboration with health economists and experienced guideline developers.

## Competing interests

The author helped to develop the current Guidelines for WHO Guidelines.

## Authors' contributions

TTTE prepared the first draft of the review and was responsible for the subsequent revisions.

## Supplementary Material

Additional file 1 Cost-effectiveness and affordability in the Guidelines for WHO Guidelines. Excerpts from the document "Guidelines for WHO Guidelines" which refer to the concepts of cost-effectiveness and affordability. Click here for file

Additional file 2 Considerations of costs/cost-effectiveness information by other agencies. Excerpts from documents from other agencies that issue guidelines which refer to the concepts of costs/cost-effectiveness Click here for file
