# Supplementary material for: Improving the use of research evidence in guideline development: 11. Incorporating considerations of cost-effectiveness, affordability and resource implications
Source: Health Res Policy Syst. 2006 Dec 5;4:23. doi: 10.1186/1478-4505-4-23 (PMC1764011; doi:10.1186/1478-4505-4-23)
Supplement: Additional file 1 — Cost-effectiveness and affordability in the Guidelines for WHO Guidelines. Excerpts from the document "Guidelines for WHO Guidelines" which refer to the concepts of cost-effectiveness and affordability. [file 1478-4505-4-23-S1.doc]

**Cost-effectiveness and affordability in the Guidelines for WHO Guidelines** [<http://whqlibdoc.who.int/hq/2003/EIP_GPE_EQC_2003_1.pdf>]

p.4 WHO needs to assess the implications for population health of any recommendation as well. This requires explicit recognition that resources to provide health interventions are limited. This involves considering the cost-effectiveness of alternative interventions, the opportunity costs of investing in one intervention versus another, the affordability of the interventions, and the feasibility of applying a set of recommendations in different settings.

p.5 The initial body of evidence to be considered in WHO guidelines will be identical to that of traditional guidelines, but WHO guidelines will need to go further, to take the second step of spelling out the implications of adopting recommendations on costs and on population health. If done adequately, this will allow decision makers in different settings to take the third step of “localizing” the guidelines to their settings, and deciding where the tradeoff between additional benefit and additional costs should be set. It will also be useful in determining what is acceptable for the end-users.

…In summary, there are 4 questions, which need to be answered in guideline development: what is efficacious? what is cost-effective? what is affordable? and what is beneficial for the population? In many instances, there will not be a single answer to all these questions. It is also to be expected that the answers will vary across countries.

p. 7

d. Making recommendations.

The second stage of WHO guideline development will spell out any tradeoffs between the cost of applying possible recommendations on a population basis, and the population health impacts. It would consist of a number of scenarios (or optimal recommendations) – perhaps in the cases of very limited resources, and unlimited resources. This would enable country decision makers to make recommendations as part of the localization process.

During the third step or the localization process, WHO should seek to provide any technical assistance necessary to countries to help them make their own recommendations. This means providing local (regional) cost-effectiveness data on the interventions concerned, data on other interventions which might be of comparable or better cost-effectiveness but which they might not yet be currently providing (to expand their policy choices within the sector), data on the resources required to carry out the interventions, including financial start-up and capital costs. This is one of the roles of GPE.

p.9 Membership of Technical Guideline Development Group…

2. Members (multidisciplinary, around 8-12 individuals) to represent:

a. stakeholders and to ensure that the right issues are identified and facilitate early buy-in for the guidelines)

Professionals

Disease experts

Primary care/public health (MOH)

End users

Patients

b. Methodologists (to ensure scientific rigour)

Practice Guideline Development

Systematic Review (at least 2)

Cost Effectiveness (1-2)

Health services/systems
