# Supplementary material for: Improving the use of research evidence in guideline development: 11. Incorporating considerations of cost-effectiveness, affordability and resource implications
Source: Health Res Policy Syst. 2006 Dec 5;4:23. doi: 10.1186/1478-4505-4-23 (PMC1764011; doi:10.1186/1478-4505-4-23)
Supplement: Additional file 2 — Considerations of costs/cost-effectiveness information by other agencies. Excerpts from documents from other agencies that issue guidelines which refer to the concepts of costs/cost-effectiveness [file 1478-4505-4-23-S2.doc]

**Considerations of costs/cost-effectiveness information by other agencies**

UK: Eccles and Mason (22), in their paper cite some illustrative guidelines in their paper which used CEA in various ways: in a qualitative manner, in a quantitative way addressing both narrow and broad questions and those which base their recommendations using decision analysis. The guidelines were variously funded by the Department of health of England and Wales and the Northern and Yorkshire R and D programme. The paper is useful in that it gives the reader an idea of the different ways that CEA can be incorporated in a guidelines, from a general consideration of costs to a decision-model driving a guideline. Currently, in the UK, guidelines are issued by NICE and about 50 or so guidelines have been issued. Its documentation of the guideline development process specifically include a chapter on Guideline Development Methods - Chapter 8: Incorporating health economics in guidelines and assessing resource impact and lists a health economist as an integral part of the guideline development group.

Scotland: SIGN recognises that, in a national health service with limited resources and ever-increasing costs, the ability to cost individual items of care and weigh these against some quantification of patient benefit is important. However, the science of economic analysis of health care is at a relatively early stage and many published studies do not meet the required methodological standard to be incorporated as part of the evidence base for a guideline. Where there is published economic evidence, this has to be identified and evaluated in a consistent manner. Health economic databases are included in the coverage of literature search and studies identified from those searches are evaluated against standard criteria. This evidence can be considered alongside clinical evidence at the considered judgement stage

The Netherlands: The Quality Assurance Programme of the Netherlands was started in 1998 and was a joint effort from (para)medical professional organizations, patient organizations, health economists and other health services researchers, to formulate evidence based guidelines with a concomitant cost-effectiveness analysis (CEA) and a strategy for implementation in daily medical practice. The project was initiated by the Dutch Ministry of Health and implemented by the National Organization for Quality Assurance in Hospitals, the WHO Collaborating Center for Quality Assurance in Health Care and the Institute for Medical Technology Assessment. There has been experience with about 31 guidelines, however this program was recently discontinued.

Australia: The Victoria state government in Australia worked with a teaching hospital to include cost-effectiveness information in respiratory and cardiology guidelines for use in the Victorian hospital networks. Australia in its NHRMC guidance for guideline developers has a specific supplementary handbook on incorporating cost-effectiveness information in guideline development.

United States. In the US, The Community Guide is being developed by the nonfederal Task Force on Community Preventive Services), whose members are
appointed by the Director of the Centers for Disease Control and Prevention (CDC). Although convened by the U.S. Department of Health and Human Services, the Task Force is an independent decision-making body. The Task Force on Community Preventive Services in its 2001 set of guidelines included systematic reviews of CEA on its guidelines but did not use the information to change the recommendation from recommended to not recommended or vice versa. The US Preventive Services Taskforce which concentrates on clinical preventive services also state that they take economic costs into consideration when possible but that these are not primary. They state that when an intervention is not recommended because of economic reasons, this is stated explicitly.

Canada: The Canadian Task Force on Preventive Services, also acknowledges the concern with rising health care costs and states that the taskforce "attempts to furnish information on cost-effectiveness of interventions" and admits that because of limited information and the appropriateness of modelling techniques to the Canadian setting , and thus, " the Task Force may only be able to describe a procedure or technology in general terms as costly and as a result tend to de-emphasize it."
